# Draft Genome Sequence of *Limnobacter* sp. Strain CACIAM 66H1, a Heterotrophic Bacterium Associated with Cyanobacteria

**DOI:** 10.1128/genomeA.00399-16

**Published:** 2016-05-19

**Authors:** Fábio Daniel Florêncio da Silva, Alex Ranieri Jerônimo Lima, Pablo Henrique Gonçalves Moraes, Andrei Santos Siqueira, Leonardo Teixeira Dall’Agnol, Anna Rafaella Ferreira Baraúna, Luisa Carício Martins, Karol Guimarães Oliveira, Clayton Pereira Silva de Lima, Márcio Roberto Teixeira Nunes, João Lídio Silva Gonçalves Vianez-Júnior, Evonnildo Costa Gonçalves

**Affiliations:** a Laboratório de Tecnologia Biomolecular, Instituto de Ciências Biológicas (ICB), Universidade Federal do Pará (UFPA), Belém, Pará, Brazil; b Universidade Federal do Maranhão (UFMA), Campus de Bacabal, Maranhão, Brazil; c Laboratório de Patologia Clínica e Doenças Tropicais, Núcleo de Medicina Tropical, Universidade Federal do Pará, Belém, Pará, Brazil; d Centro de Inovações Tecnológicas (CIT), Instituto Evandro Chagas (IEC), Ananindeua, Pará, Brazil

## Abstract

Ecological interactions between cyanobacteria and heterotrophic prokaryotes are poorly known. To improve the genomic studies of heterotrophic bacterium-cyanobacterium associations, the draft genome sequence (3.2 Mbp) of *Limnobacter* sp. strain CACIAM 66H1, found in a nonaxenic culture of *Synechococcus* sp. (cyanobacteria), is presented here.

## GENOME ANNOUNCEMENT

The genus *Limnobacter* comprises Gram-negative, non-spore-forming, strictly aerobic, and slightly curved rods, which are motile by a single polar flagellum. Species are able to use thiosulphate as an additional energy source (1, 2). *Limnobacter* spp. were detected by genetic screening as phenol degraders and thus may act in biodegradation processes (3). Cyanobacteria are known producers of several compounds of biotechnological interest (4, 5) and generally require the presence of heterotrophic aerobic bacteria for growth (6). However, the ecological interactions between photosynthetic cyanobacteria and heterotrophic prokaryotes are poorly known. To improve the genomic study of heterotrophic bacterium-cyanobacterium associations, we obtained the draft genome of *Limnobacter* sp. strain CACIAM 66H1 from the sequencing of a nonaxenic culture of *Synechococcus* sp. strain CACIAM 66 (Biosample SAMN04546176), which was isolated from a water sample from the Tucuruí hydroelectric power station reservoir (3°49′55″S, 49°38′50″W) in Pará, Brazil.

After DNA extraction of the cyanobacterial culture, three sequencing runs were performed on the GS FLX 454 (Roche Life Sciences) platform using nonpaired libraries, and one sequencing run was carried out on the Illumina MiSeq platform with a paired-end library with 150-bp read length. All the raw reads obtained were quality filtered with a minimum Phred score of 20, resulting in 3,291,965 (~92 Gb) filtered reads. A coassembly of all reads was performed by gsAssembler (Newbler version 2.9), with the following parameters: minimum overlap of 20 bp, minimum overlap identity of 80%, and heterozygote mode and extend low-depth overlap options on. MaxBin 2.0 (7) was used to bin the assembled sequences. To classify taxonomically the obtained bins, we performed BLASTP for each bin in the sequences containing hidden Markov models for essential genes identified by MaxBin 2.0 against the NCBI nonredundant database. The results were visualized on MEGAN 5.11.3 (8).

The binned genome of *Limnobacter* sp. CACIAM 66H1 was annotated by the Prokaryotic Genome Annotation Pipeline (9). The genome has 108 scaffolds, a total length of 3,217,571 bp, *N*_50_ of 49,750 bp, and G+C content of 52.45%. The annotation pipeline predicted 3,076 protein-coding sequences, 4 rRNA genes, 40 tRNA genes, and 3 noncoding RNAs. The genome of *Limnobacter* sp. CACIAM 66H1 possesses 104 of the 107 essential genes used as models by MaxBin 2.0, with duplication of ribosomal L6, allowing completeness near 96.3%.

The draft genome sequence we obtained should be useful for a better understanding of the heterotrophic bacterium-cyanobacterium association, and for elucidating issues about bacterial growth and the production of compounds of biotechnological interest.

### Nucleotide sequence accession numbers.

This whole-genome shotgun project has been deposited at DDBJ/ENA/GenBank under the accession no. LUJK00000000. The version described in this paper is version LUJK01000000.
